# Global Increase of Antibiotic Resistance Genes in Conjugative Plasmids

**DOI:** 10.1128/spectrum.04478-22

**Published:** 2023-03-22

**Authors:** Xiaolong Wang, Hanhui Zhang, Xiang Long, Ximing Xu, Hongqiang Ren, Daqing Mao, Pedro J. J. Alvarez, Yi Luo

**Affiliations:** a College of Environmental Science and Engineering, Ministry of Education Key Laboratory of Pollution Processes and Environmental Criteria, Nankai University, Tianjin, China; b State Key Laboratory of Pollution Control and Resource Reuse, School of the Environment, Nanjing University, Nanjing, China; c School of Statistics and Data Science, Nankai University, Tianjin, China; d School of Medicine, Nankai University, Tianjin, China; e Department of Civil and Environmental Engineering, Rice University, Houston, Texas, USA; Brown University

**Keywords:** conjugative plasmid, antibiotic resistance genes, global increase, antibiotics, transmission

## Abstract

Antibiotic resistance is propagating worldwide, but the predominant dissemination mechanisms are not fully understood. Here, we report that antibiotic resistance gene (ARG) abundance in conjugative plasmids that are recorded in the National Center for Biotechnology Information (NCBI) RefSeq plasmid database is increasing globally, which is likely a key factor in the propagation of resistance. ARG abundance in plasmids increased by 10-fold on a global scale from the year 2000 to the year 2020 (from 0.25 to 2.93 ARG copies/plasmid), with a more pronounced increase being observed in low-to-middle income countries. This increasing trend of plasmid-borne ARGs was corroborated by bootstrap resampling from each year of the NCBI RefSeq plasmid database. The results of a correlation analysis imply that if antibiotic consumption keeps growing at the current rates, a 2.7-fold global increase in the ARG abundance of clinically relevant plasmids may be reached by 2030. High sequence similarities of clinically relevant, conjugative plasmids that are isolated both from clinics and from the environment raise concerns about the environmental resistome serving as a potential ARG maintenance reservoir that facilitates transmission across these ecological boundaries.

**IMPORTANCE** Antibiotic resistance propagation is a significant concern due to its projected impacts on both global health and the economy. However, global propagation mechanisms are not fully understood, including regional and temporal trends in the abundance of resistance plasmids that facilitate antibiotic resistance gene (ARG) dissemination. This unprecedented study reports that ARG abundance in the conjugative plasmids that are recorded in the National Center for Biotechnology Information (NCBI) database and harbor ARGs is increasing globally with antibiotic consumption, especially in low-to-medium income countries. Through network and comparative genomic analyses, we also found high sequence similarities of clinically relevant conjugative resistance plasmids that were isolated from clinical and environmental sources, suggesting transmission between these ecological boundaries. Therefore, this study informs the One Health perspective to develop effective strategies by which to curtail the propagation of plasmid-borne antibiotic resistance.

## INTRODUCTION

Plasmids are extrachromosomal genetic elements that generally endow bacteria with peripheral functions to deal with stress, such as antibiotic resistance (1). 50 percent of the bacteria in nature contain one or more plasmids (2), whereas the percentage of clinical bacteria that contain plasmids is even higher; for example, plasmid pO157 was found in over 99% of clinical enterohemorrhagic E. coli O157:H7 isolated from humans (3). Furthermore, plasmid-encoded antibiotic resistance genes (ARGs) that protect bacteria from commonly used antibiotics are frequently reported (4), including those that confer resistance to penicillin (5), cephalosporins (6), macrolides (7), tetracyclines (8), aminoglycoside (9), quinolones (10), and even to the last-lines-of-defense, namely, tigecycline and colistin (11, 12). However, the manner in which the relative abundance of plasmid-borne ARGs is evolving globally as a function of annual antibiotic use is unknown. This is an important knowledge gap in the discernment of the predominant resistance propagation mechanisms. If these patterns differ significantly between high-income and low-to-middle-income countries, research in this area could inform the development of appropriate regional policies and measures by which to curtail the global propagation of antibiotic resistance.

The number of completely sequenced plasmids has increased exponentially over the past 20 years due to the advent of high-throughput DNA sequencing. As of April of 2021, 27,938 fully sequenced plasmids could be retrieved from the National Center for Biotechnology Information (NCBI) RefSeq plasmid database, including information regarding the isolation dates and countries of the plasmids. Global data are also now available for antibiotic consumption, which differs greatly between low-income and middle-income countries versus high-income countries (13). Analyzing these data sets enables the assessment of the temporal and spatial distribution of the recorded plasmids (including those that transmit ARGs) as a function of antibiotic consumption. This represents an opportunity to advance the understanding of the propagation dynamics of plasmid-borne antibiotic resistance at a global scale as well as to predict regional trends that are associated with various policies of antibiotic administration.

Clinically relevant plasmid-borne ARGs (e.g., *bla*_NDM-1_, *bla*_KPC2_, and mcr-1) are frequently detected in the environment, including in river sediments (8, 14), river water (15), seawater (16), soil (17), and airborne particle matter (18). However, a clear etiology between environmental ARGs and clinical resistance has not been established (19), thereby precluding the assessment of the importance of the environmental resistome as a potential ARG maintenance, amplification, and transmission reservoir. Nevertheless, the NCBI database includes plasmid sources (clinic or environment), which could help develop a more holistic etiological perspective between clinical and environmental antibiotic resistance (e.g., through nucleotide sequence comparisons [20]) to inform appropriate approaches by which to curtail the dissemination of antibiotic resistance.

In the present study, we retrieved and analyzed completely sequenced plasmids that are available in the NCBI database, which includes 27,938 plasmids that were isolated from various habitats worldwide from 2000 to 2020. We tracked the temporal trend of plasmid-borne ARG abundance during each year from 2000 to 2020, and this was corroborated by bootstrap resampling. We also considered the global antibiotics consumption data set (21), which differentiates use patterns between high-income countries and low-income and middle-income countries. Combining these data sets facilitated the development of a regression model to predict the ARG abundance in plasmids as a function of antibiotic consumption. Network and comparative genomic analyses were also conducted to assess the potential transmission of plasmid-borne ARGs between environmental and clinical settings, which is important both to inform the One Health perspective and in the development of effective strategies by which to curtail the propagation of plasmid-borne antibiotic resistance.

## RESULTS AND DISCUSSION

### Plasmid-borne ARG abundance from the NCBI database is increasing globally.

Previous analyses of increases in antibiotic resistance have focused primarily on the increasing ratios of resistant pathogens rather than on the ARG abundance in mobile genetic elements that serve as transmission vectors (22, 23). Therefore, global-scale trends in plasmid-borne ARG abundance have been overlooked.

In the present study, 27,938 fully sequenced plasmids that were recorded globally were retrieved from the NCBI RefSeq plasmid database. Based on their mobility, plasmids were classified into three categories (i.e., conjugative, mobilizable, and nonmobilizable). Both conjugative and mobilizable plasmids are capable of disseminating ARGs from donor to recipient bacteria, but conjugative plasmids carry all of the genes that are needed for self-transfer, whereas mobilizable plasmids lack the genes that are required for mating pair formation. Thus, conjugative plasmids have higher transfer frequencies (10^−3^ to 10^−1^), which are generally at least 1 order of magnitude higher than those of mobilizable plasmids (24). This database included 7,132 conjugative plasmids, 8,443 mobilizable plasmids, and 12,363 nonmobilizable plasmids. The number of plasmids in this database increased 123-fold (from 227 to 27,938) over the past 2 decades. After alignment against the Structured Antibiotic Resistance Genes (SARG) database, a total of 38,151 ARGs were identified from all of the collected plasmids, and 90.2% of the total ARGs were validated by DeepARGs. Conjugative plasmids accounted for only 25.5% of the total plasmids, but they carried 47.8% of the total plasmid-borne ARGs.

**FIG 1:**
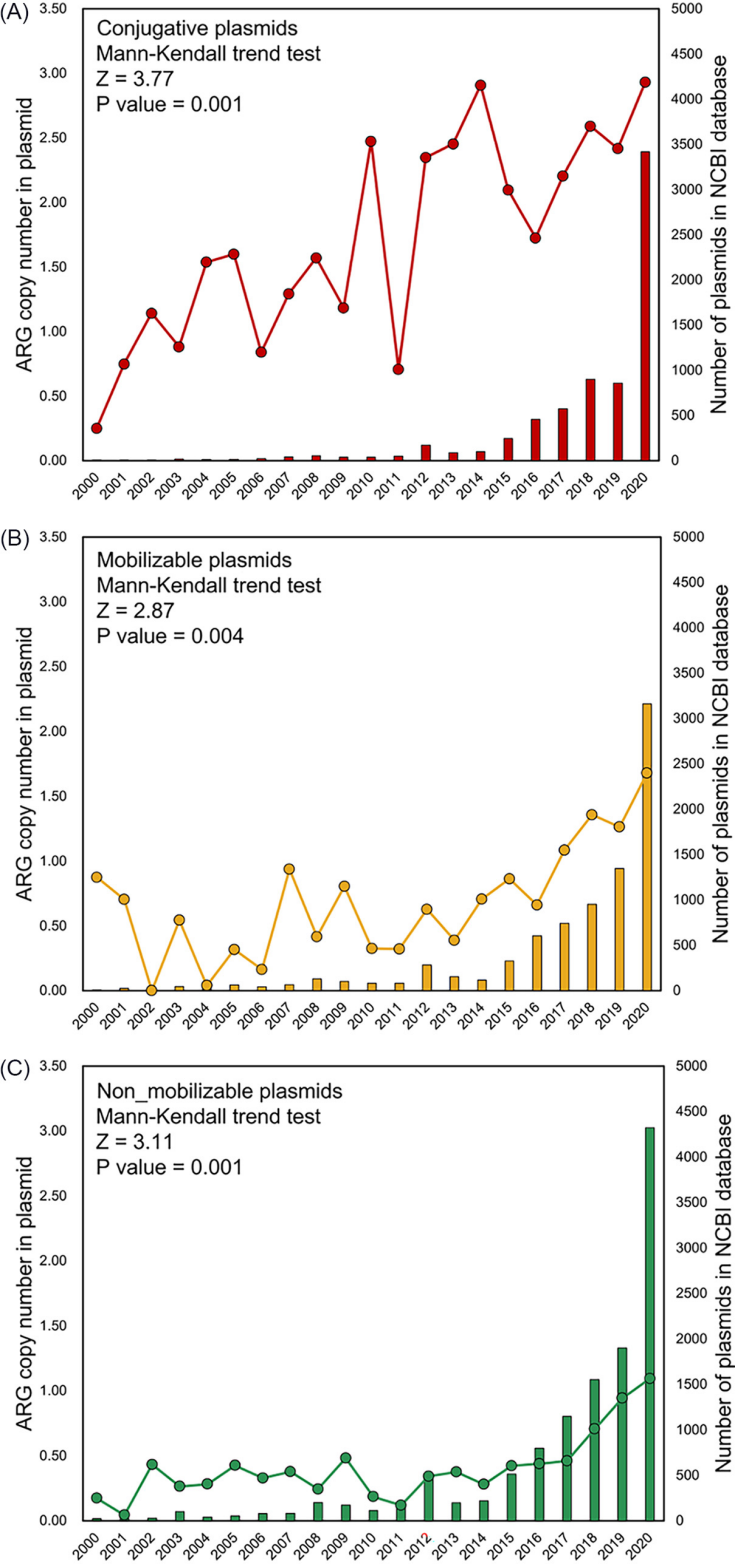
Plasmids harboring ARGs from the year 2000 to the year 2020 that were retrieved from the NCBI RefSeq plasmid database. The abundance of plasmid-borne ARGs exhibited a significant, increasing trend for (A) conjugative plasmids, (B) mobilizable plasmids, and (C) nonmobilizable plasmids. A Mann-Kendall trend test *z* value of >1.96 indicates that the increasing trend is statistically significant (*P* < 0.05).

**FIG 2:**
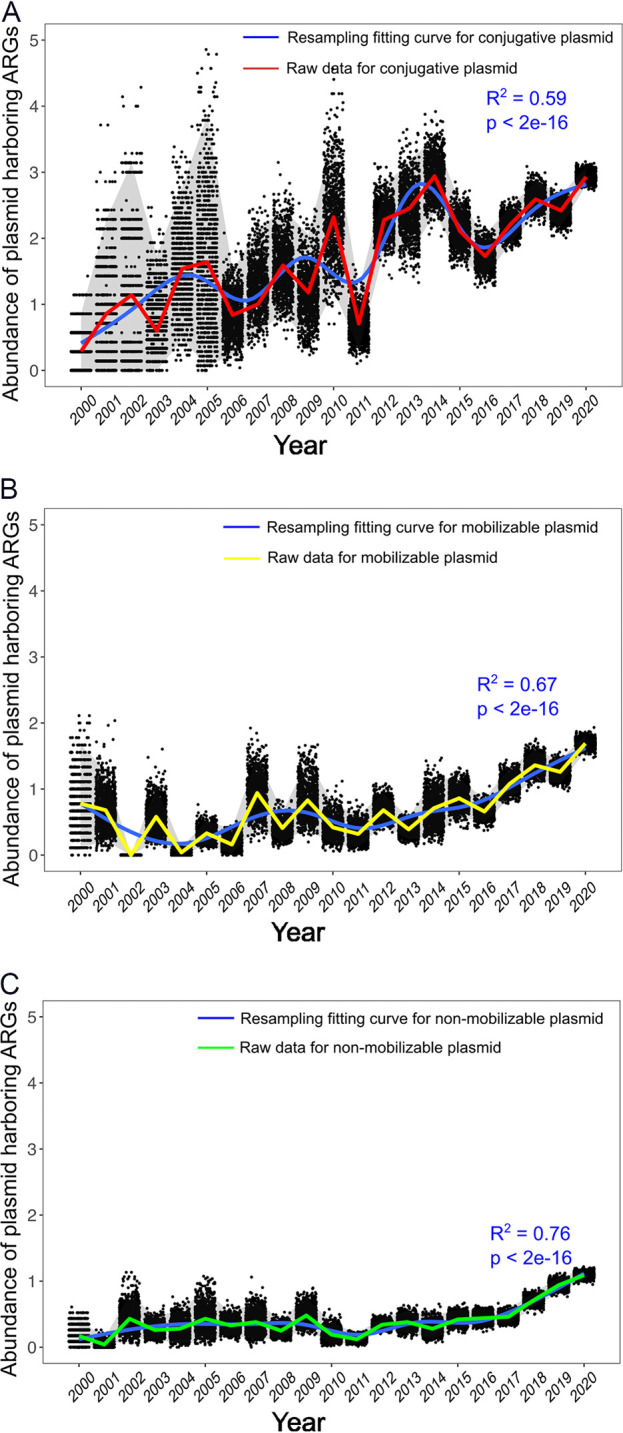
Bootstrap resampling fitting curve of the abundance of plasmid-borne ARGs. (A) Conjugative plasmids. (B) Mobilizable plasmids. (C) Nonmobilizable plasmids. Each dot represents the plasmid-borne ARG abundance that was calculated from each resampled trial. The shading depicts a 95% confidence interval. The blue line represents the bootstrapping sampling fitting curve. The R^2^ value and the *P* value were used to evaluate goodness-of-fit. The red, yellow, and green lines represent the raw data of the conjugative plasmids, mobilizable plasmids, and nonmobilizable plasmids, respectively. The agreement of the raw data with the fitted values is 87.9% for the conjugative plasmids, 88.7% for the mobilizable plasmids, and 94.1% for the nonmobilizable plasmids.

To characterize the temporal trends in the abundance of plasmids harboring ARGs, the number of plasmid-borne ARGs in the NCBI RefSeq database was determined for each year from 2000 to 2020. The ARG abundance in the conjugative plasmids exhibits a significant, increasing trend from 2000 to 2020 (Mann-Kendall trend test, *z* = 3.77, *P* = 0.0001), indicating a 1,072% increase from 0.25 to 2.93 ARG copies per plasmid (Fig. 1A). The ARG abundance in the mobilizable plasmids and in the nonmobilizable plasmids also increased significantly over this time period, although to a lesser extent (i.e., by 96%, from 0.85 to 1.67 copies per plasmid, in the mobilizable plasmids [Fig. 1B] and by 920%, from 0.10 to 1.02 copies per plasmid, in the nonmobilizable plasmids [Fig. 1C]). Variations in the sources and numbers of sequenced plasmids for different years might be influenced by various research interests and advances in sequencing technology, thereby representing potential confounding effects. Therefore, a bootstrap resampling approach was applied to both address the heterogeneity across the years annual and enhance our statistical inferences. This analysis showed a clearly increasing trend in the simulated plasmid-borne ARG copy numbers (95% confidence interval) from 2000 to 2020 (Fig. 2), which is in excellent agreement with the raw data. The agreement of the raw data with the fitted values is 87.9% for the conjugative plasmids (Fig. 2A), 88.7% for the mobilizable plasmids (Fig. 2B), and 94.1% for the nonmobilizable plasmids (Fig. 2C).

### The increasing plasmid-borne ARG abundance is significantly correlated with an increasing trend of antibiotic consumption, particularly in low-to-middle income countries.

**FIG 3:**
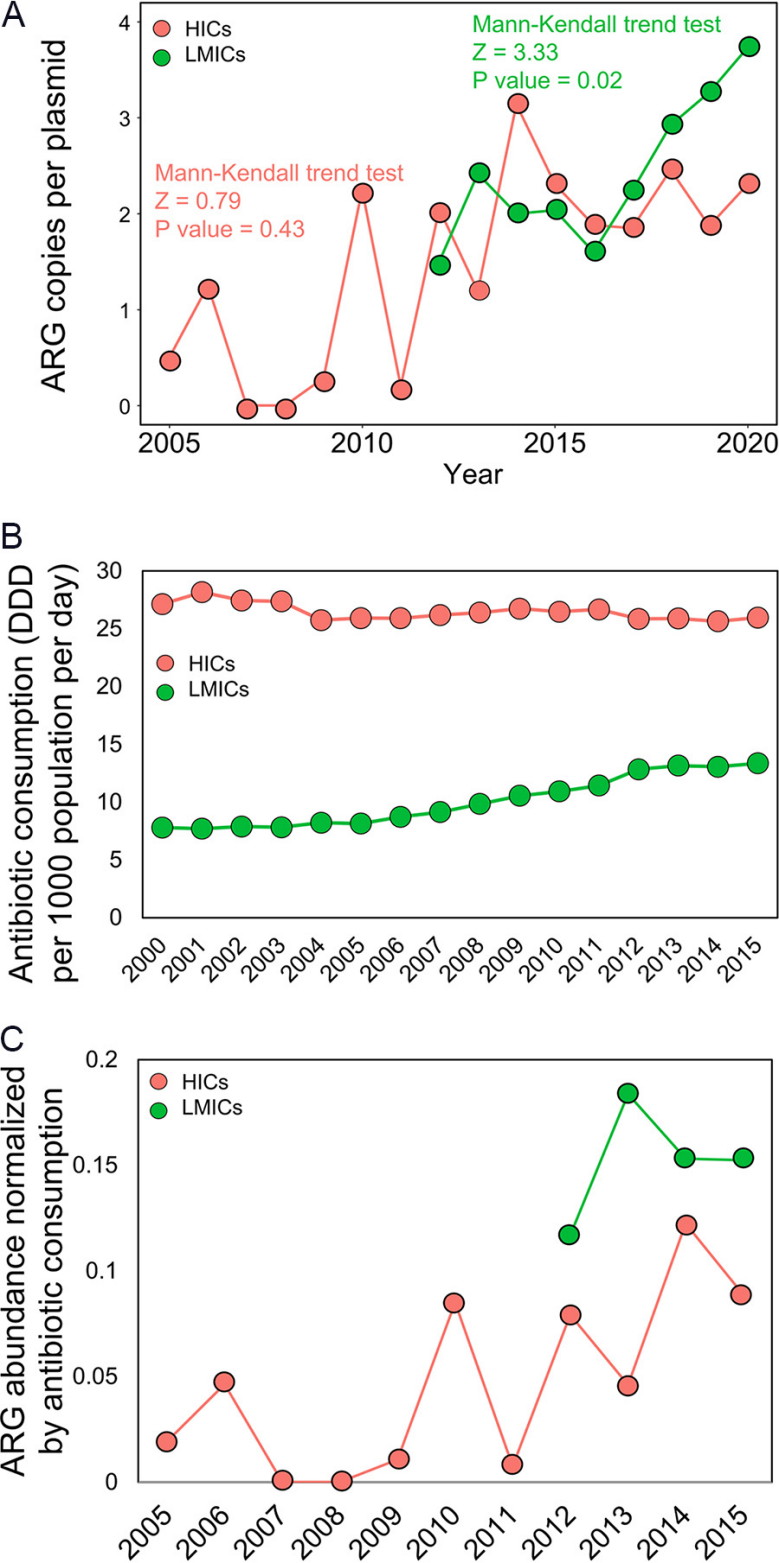
Increase in abundance of plasmid-borne ARGs with antibiotic consumption in high-income countries and low-to-middle income countries. (A) Increase of plasmid-borne ARG abundance in high-income countries and low-to-middle income countries. A Mann-Kendall trend test *z* value of >1.96 indicates a significant, increasing trend (*P* < 0.05). It was also calculated that *z* = 3.33 (*P* = 0.02) in the low-to-middle income countries and that *z* = 0.79 (*P* = 0.43) in the high-income countries. (B) Antibiotic consumption (DDD per 1,000 population, per day) in the high-income countries and in the low-to-middle income countries. (C) The ARG abundance, normalized by the antibiotic consumption, in the high-income countries and in the low-to-middle income countries.

A growing use of antibiotics for both human applications and animal agriculture, as well as their environmental residues, have exerted selective pressure for ARG proliferation and dissemination among bacteria (25, 26). Thus, we explored whether the increase in ARG abundance in conjugative plasmids was correlated with the global increase in antibiotic consumption. We found a significant correlation (Spearman’s ρ = 0.62, *P = *0.01). In general, the plasmid-borne ARG abundance increased from 2000 to 2020 in both low-to-middle income countries (LMICs) and high-income countries (HICs) (Fig. 3A), with a more pronounced increasing trend in LMICs, even though their antibiotic consumption (measured in DDDs per 1,000 inhabitants per day) was much lower (Fig. 3B).

The large contrast of ARG abundance (Fig. 3A) and antibiotic consumption (Fig. 3B) between LMICs and HICs is remarkable. When ARG abundance values in plasmids are normalized to the global antibiotic consumption (plasmid-borne ARG abundance/DDDs per 1,000 inhabitants per day), it becomes clear that the normalized ARG abundance values are significantly higher in LMICs than in HICs (Fig. 3C). This suggests that LMICs have a higher relative abundance of plasmid-borne ARGs, even if their antibiotic consumption is similar to that observed in HICs, which could be partly attributable to the misuse and abuse of antibiotics in LMICs. The inappropriate use of antibiotics has been proposed as a key driver in the propagation of antibiotic resistance (27), and it is becoming a global threat (28). Children in LMICs have been reported to receive an average of 25 antibiotic prescriptions during their first 5 years of life (29), which is significantly higher than the 2 average antibiotic prescriptions per year in HICs (30).

**FIG 4:**
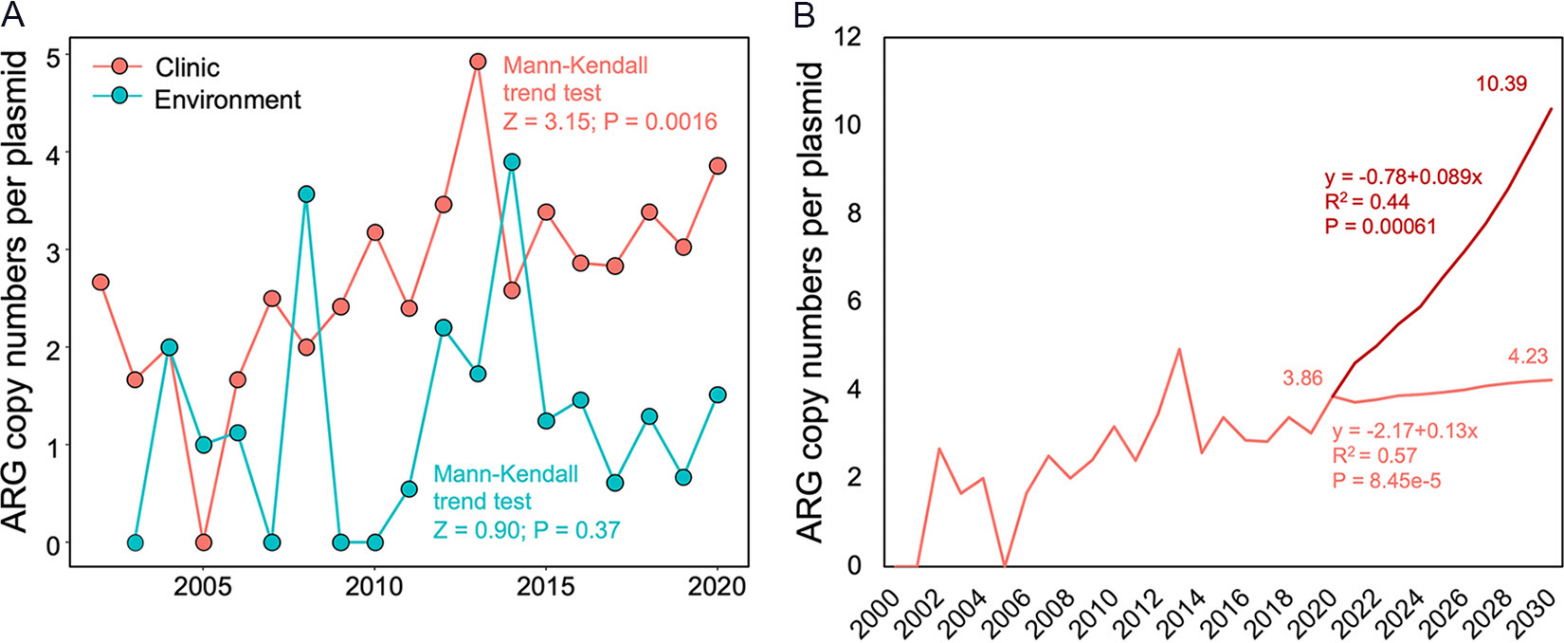
Increase in abundance of plasmid-borne ARGs in the clinic and in the environment. (A) The increasing values of the plasmid-borne ARGs in clinical or environmental isolates from 2000 to 2020. (b) Projected plasmid-borne ARGs in the clinic from 2021 to 2030. The projections are based on the global antibiotic consumption for two scenarios: (i) all countries maintain their current application rate constants and (ii) the consumption of all countries continues to change at the current compounded annual growth rates.

Clinics are hot spots of human antibiotic usage, underscoring the need for stewardship of appropriate antibiotic prescription. In this study, plasmid-borne ARGs displayed a significant increase of 131.1%, from 1.67 to 3.86 copies/plasmid, in clinical isolates (Mann-Kendall trend test, *z* = 3.15, *P* = 0.0016), compared to a smaller increase of 50.0%, from 1 to 1.50 copies/plasmid, in environmental samples from 2000 to 2020 (Fig. 4A). Based on the positive correlation between antibiotic consumption and plasmid-borne ARGs isolated from clinics (Spearman’s ρ = 0.84, *P = *4.30 × 10^−5^), we infer that if antibiotic consumption is kept at the current annual growth rates, global plasmid-borne ARGs will increase by 269% by the year of 2030 (Fig. 4B). However, if antibiotic consumption is kept constant at the current levels, global plasmid-borne ARGs will increase by only 110% (Fig. 4B).

### A DNA sequence homology assessment suggests potential resistance transmission between the environment and the clinic.

Pathogenic bacteria carrying clinically relevant ARGs are regularly inadvertently discharged to the environment, and these ARGs may become part of the environmental resistome (31–33). A growing concern is the potential for the maintenance and proliferation of such ARGs in the environment, with eventual transmission back to bacteria that cause human infections that are difficult to treat. For example, there are reports of patients who were infected with New Delhi beta-metalloelastase (NDM-1)-positive bacteria and did not have a history of clinical exposure, suggesting infection by pathogens (expressing extended-spectrum β-lactamases) that are circulating in the environment (34). Another study provided evidence for the horizontal transfer of ARGs between environmental bacteria and clinical pathogens, based on DNA sequencing using a high-throughput metagenomic approach (20). Similarly, a recent study using the SourceTracker algorithm inferred the propagation of ARGs from farm bacteria to the gastrointestinal microbiota of farm workers after temporal exposure (35).

The increase in the abundance of plasmid-borne ARGs could enhance the transmission of resistance between the environment and the clinic. Although plasmids are known to harbor and transfer ARGs, their criticality in the transmission of resistance between the environment and the clinic has not been established. This study offers converging circumstantial evidence that conjugative plasmids do play a critical role in the transmission of ARGs across these ecological boundaries. This includes the fact that clinical and environmental plasmid-borne ARGs increased synchronously with antibiotic consumption in both HICs and LMICs as well as sequence-based homology between plasmids that were isolated from clinical and environmental bacteria.

**FIG 5:**
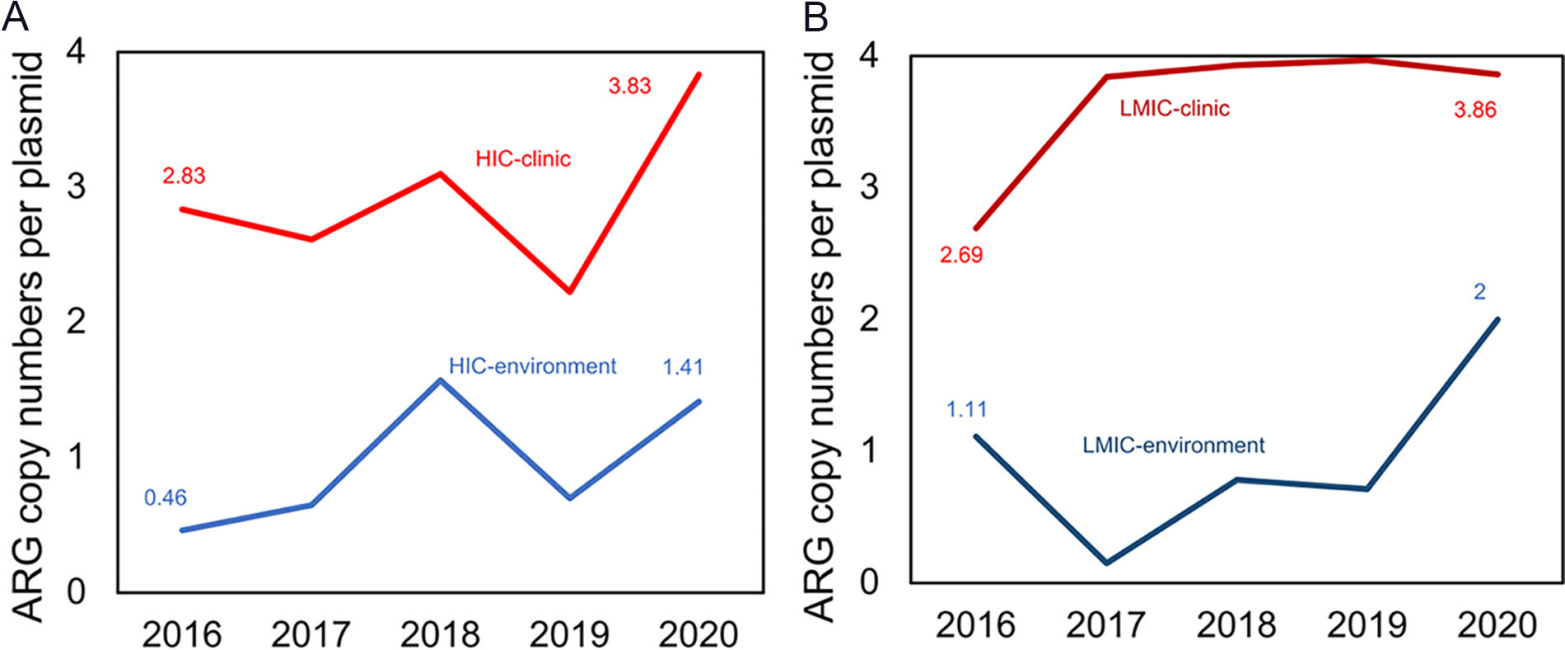
Increasing abundance of plasmid-borne ARGs in the clinic and environment from 2016 to 2020. (A) High-income countries. (B) Low-and middle-income countries.

Regarding the first line of evidence, clinical plasmid-borne ARGs in HICs exhibited a 35.3% increase from 2016 to 2020, whereas environmental plasmid-borne ARGs increased by 206% from 2016 to 2020 (Fig. 5A). Similarly, in LMICs, clinical plasmid-borne ARGs exhibited a 43.5% increase from 2016 to 2020, whereas environmental plasmid-borne ARGs increased by 81.8% from 2016 to 2020 (Fig. 5B).

We used sequence-based homology to analyze plasmids harbored by different bacteria that were isolated from clinics and from the environment in order to assess the potential propagation of ARGs across these ecological boundaries. In our collected plasmids, 2,420 conjugative plasmids were categorized into the “clinic” source category, and 882 conjugative plasmids were categorized into the “environment” source category, based on the plasmid isolation source. After plasmid classification, based on plasmid incompatibility groups, a total of 48 plasmid incompatibility groups (PIGs) were identified in the “clinic” source category, among which 30 PIGs were coshared with the “environment” category. Among those 30 coshared PIGs, IncF, IncN, and IncX3 were prevalent and accounted for 48.3%, 8.6%, and 3.1%, respectively, of the coshared plasmids. The homologous plasmids that are coshared by both categories (with a nucleotide identity of >95% and a coverage of >90%) account for 37.5% of the total plasmids from environmental sources and 8.7% from clinical sources.

**FIG 6:**
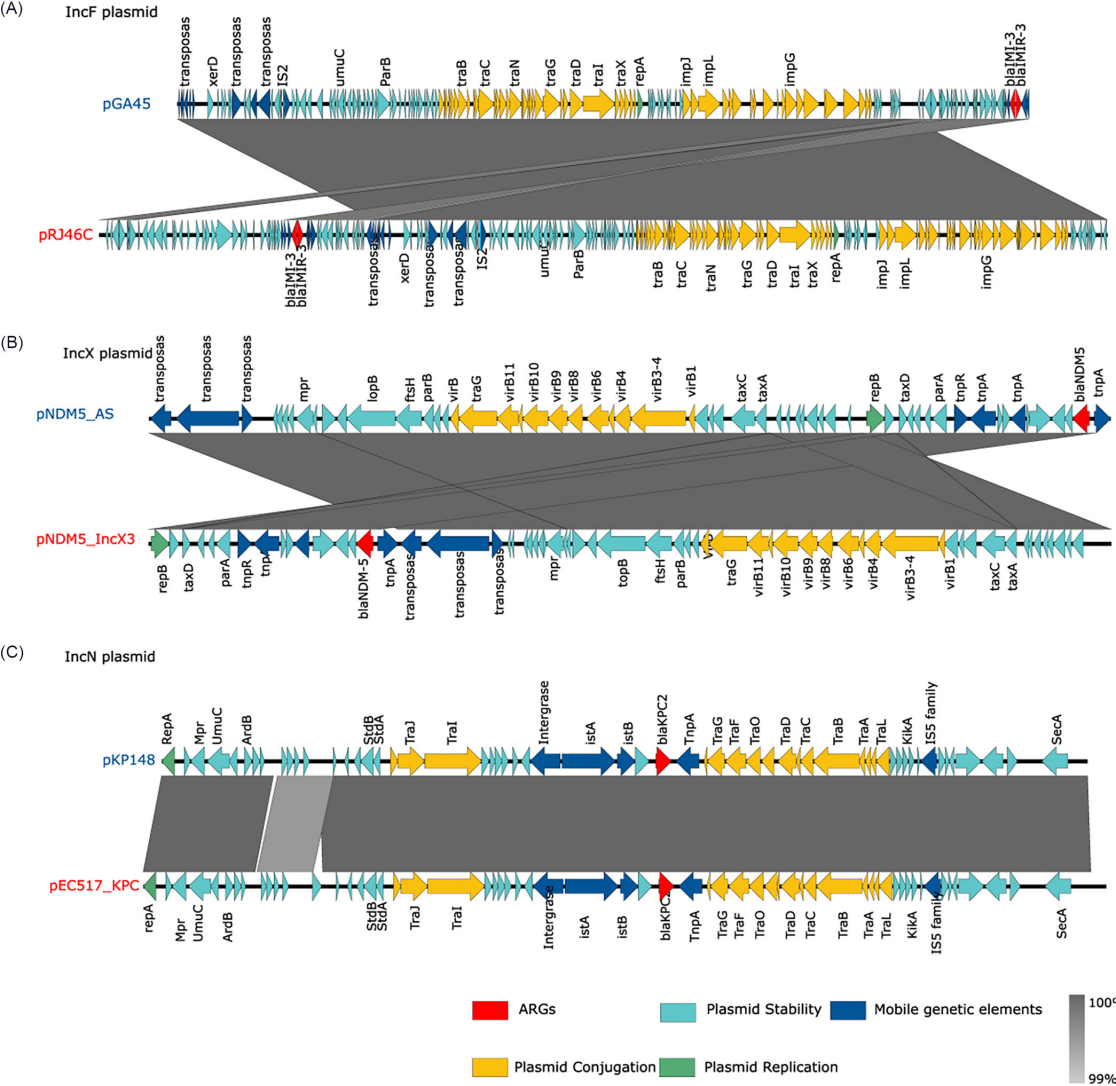
The high sequence similarity (>99%) of plasmids from environmental and clinical sources. The high sequence similarities suggest that these plasmids have common ancestors. The panels show these similarities between (A) the IncN pKP148 plasmid from the “environment” source category and the clinic-derived pEC517_KPC plasmid, (B) the IncF pGA45 plasmid from the “environment” category and the pRJC46 plasmid from the “clinic” category, and (C) the IncX3 pNDM5_AS plasmid from the “environment” category and the pNDM_IncX3 plasmid from the “clinic” category. Blue or red font indicates the plasmids that were isolated from the environment or the clinic, respectively. The shaded regions (gray color) depict homologous gene clusters with a similarity value of >99%.

Pairwise comparisons of plasmids were conducted, and representative plasmids are presented in Fig. 6. The IncN pKP148 plasmid was isolated from the environment, and the pEC517_KPC plasmid was isolated from a clinical source, but they shared 99.9% identity and 99% coverage of plasmid sequences (Fig. 6A). Similar homologies were found in IncF plasmids (pGA45 and pRJ46C) (Fig. 6B) and in IncX3 plasmids (pNDM_AS and NDM5_IncX3) (Fig. 6C). The perfect nucleotide sequence match of plasmids isolated from clinical and environmental sources provides evidence for clinically relevant plasmids circulating in the environment, thereby reflecting interconnectivity between the environmental and clinical resistomes. Note that these data are insufficient to establish that the horizontal transfer of plasmids is occurring between clinical and environmental bacteria, as some plasmids might be harbored by the original hosts that were discharged from a clinic to the environment.

**FIG 7:**
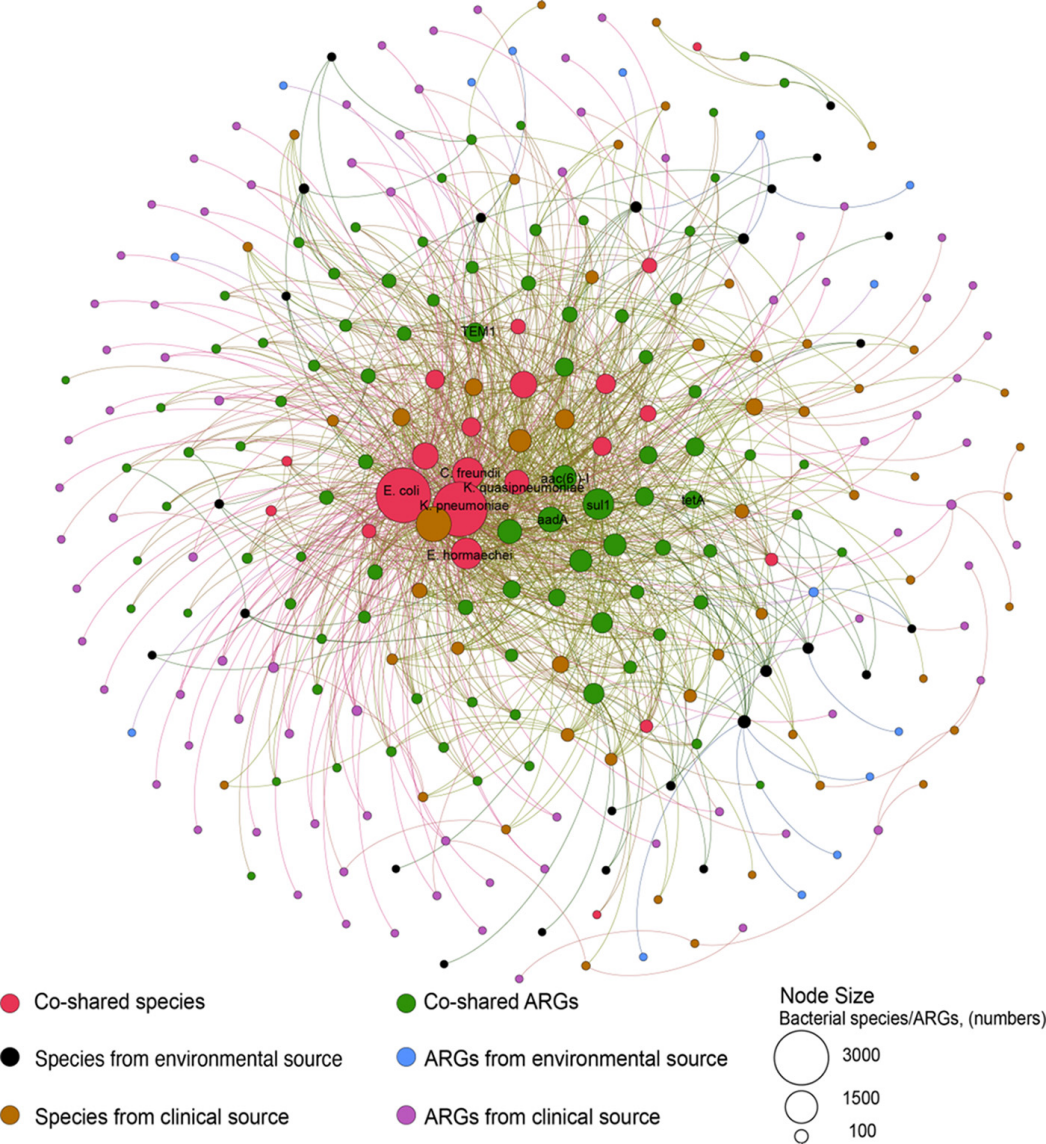
The cooccurrence of the plasmid-borne ARGs and the bacterial host of the corresponding plasmid, isolated either from a clinic or from the environment. The network lines connect the plasmid-borne ARGs to their bacterial hosts, and the sizes of the nodes (circles) represents the abundance of the ARGs or bacterial species, each of which is colored, according to its source (clinic or environment). High connectivity (with a larger size of the nodes) represents coshared ARGs (*sul1*, *tem1*, and *aadA*) and host species (E. coli, K. pneumoniae, and C. freundii) between the clinic and the environment, suggesting that those coshared ARGs were transmitted among those coshared bacterial species. The top five coshared ARGs and species are labeled in the figure. The coshared pathogen species connecting number of ARG subtypes is shown in Table S2. The raw data for this figure are presented in Table S3.

We identified the plasmid-borne ARGs and the host bacteria that were coshared between the “clinic” and “environment” sources to assess potential transmission across these ecological boundaries. To assess the potential transmission of ARGs across these ecological boundaries, we constructed a theoretical network connecting plasmid-borne ARGs and corresponding hosts (Fig. 7). A total of 89 ARG subtypes were coshared between the clinic and the environment, of which the coshared ARG subtypes accounted for 84.8% of the total plasmids from environmental sources and 52.4% of the total plasmids from clinical sources. Those coshared ARGs were carried by 36 bacteria genera and 89 bacteria species (Fig. 7). This relatively high cooccurrence of ARGs and bacteria between the clinic and the environment suggests the transmission of ARGs among the coshared bacteria (Fig. 7). Furthermore, the cooccurrence of plasmid-borne ARGs and bacteria between the clinic and the environment suggests that clinically relevant plasmids might be exchanged with pathogens (e.g., Klebsiella pneumoniae, Klebsiella oxytoca, Klebsiella variicola, Escherichia coli, Enterobacter cloacae, Enterobacter hormaechei, and Salmonella enterica) (Table S3).

The database used in the present study compiles sequences that were voluntarily reported, which may be influenced by research funding, contemporary research interests, advances in sequencing technology, and other potential confounding factors. Therefore, a bootstrap resampling approach was used to reduce the annual data heterogeneity. The random sampling fitting curve exhibited excellent agreement with the raw data, corroborating our conclusion that plasmid borne ARGs abundance is increasing over time (from 2000 to 2020). Therefore, our analysis infers a highly significant global increase in the abundance of conjugative plasmids that harbor ARGs, which is correlated with a global increase in antibiotic consumption. Accordingly, this study highlights the need to encourage (i) the appropriate prescription and responsible use of antibiotics in LMICs, (ii) a reduction of antibiotic consumption in HICs, and (iii) the interception of common environmental transmission routes of plasmid borne ARGs so as to mitigate the global propagation of clinically relevant antibiotic resistance.

## MATERIALS AND METHODS

### Batch download of plasmid sequences from the NCBI RefSeq plasmid database.

A total of 27,938 complete plasmid genomes were downloaded from the NCBI RefSeq database (ftp://ftp.ncbi.nlm.nih.gov/refseq/release/plasmid/) in April of 2021, in the GenBank and FASTA formats. These plasmids were isolated from 76 countries, worldwide, spanning over 20 years (from 2000 to 2020). The plasmid information, including the bacterial host of the plasmid, isolation date, country, and isolation source (classified as either the clinic category or the environment category) were extracted from the GenBank files via the fields of “organism”, “collection_date”, “country”, and “isolation_source”, using a self-written Python script. All of the retrieved plasmid sequences were used for subsequent analyses, including plasmid classification, ARG identification, and statistical analyses. All of the plasmid information is shown in Table S1.

### Plasmid classification and ARG annotation.

The plasmids were classified into three categories (conjugative, mobilizable, and nonmobilizable) using PlasCad (4), according to the protein machinery associated with the DNA transfer, including relaxase, the type IV coupling protein (T4CP), and the type IV secretion system (T4SSs). A plasmid containing relaxase, T4CP, and T4SSs is considered to be a conjugative plasmid, whereas one encoding only a relaxase is called a mobilizable plasmid. Genes in plasmids were annotated as ARGs when their BLASTN alignments exceeded 90% identity as well as 80% of the length of the genes in the SARG database (36). We also analyzed the SARG database using the DeepARGs deep learning approach (37) to validate these ARGs. Replicon typing was also performed using the conventional *in silico* tool PlasmidFinder with the default parameters (identity of >90% and coverage of >60%) (38).

The ARG abundance (i.e., the ARG number normalized to the total number of plasmids reported in the NCBI database) was determined to discern temporal and spatial trends in the ARG abundance in plasmids.

### Collection of the antibiotic consumption data set.

The data on antibiotic consumption were obtained from the IQVIA MIDAS data set, which is now publicly available (https://resistancemap.cddep.org/AntibioticUse.php) (21). This data set estimates the consumption of antibiotics sold in retail and hospital pharmacies, based on national surveys. Antibiotic consumption, measured in terms of defined daily doses (DDDs) per 1,000 inhabitants, per day, in high-income countries or low-income and middle-income countries, was calculated using population estimates from the World Bank DataBank (https://databank.worldbank.org/source/population-estimates-and-projections).

### Definitions of low-to-middle income countries and high-income countries.

Country income classifications (low-to-middle income countries and high-income countries) were based on their World Bank income classifications. Briefly, the classifications are based on the gross national income (GNI) per capita in terms of current USD. If the GNI per capita is greater than 12,535, the country was classified as a high-income country. Otherwise, the country was classified as a low-to-middle income country.

### Comparative genomic analyses of plasmid sequences between the clinic and the environment.

Comparative analyses were performed to analyze the similarity and the homology of the sequenced plasmids that were isolated from environmental or clinical sources. Plasmids that were isolated from the environment or from the clinic were used for pairwise sequence alignments (BLASTN). These plasmids, with high homology (nucleotide identity of >95% and coverage of >90%), were aligned using the Mauve tool with the default settings. Subsequently, the annotation results of both the analyzed plasmids and the alignment were visualized in R, using the genoPlotR package.

### Bootstrap resampling.

A bootstrap resampling approach was applied to address the potential confounding effects and the heterogeneity in the year-to-year data. For the plasmid data coming from each year, we considered the total numbers of plasmids from each year with replacement, and we then calculated the plasmid-borne ARG abundance, based on the random sampling results. These trials were repeated 1,000 times for each year. Using the R package *mgcv*, a generalized additive model (GAM) was conducted to identify the best-fit curve for the estimation of the trend of plasmid-borne ARGs abundance over time, based on the random sampling results. The fitting curve was plotted with a 95% confidence interval using the R package *ggplot2*. R^2^ values and *P* values were used to evaluate the goodness-of-fit. Finally, the Pearson correlation coefficient was used to assess the similarity between the raw data and the fitted values.

### Statistical analysis.

To discern the cooccurrence of the plasmid-borne ARGs and the bacterial hosts of corresponding plasmids, a network connecting the ARGs and the bacterial hosts of the plasmids was constructed, based on the physical linkage of the plasmid-borne ARGs and their bacterial hosts in the clinic and in the environment. The Gephi software package was used for the network visualization and manipulation. The locations of the plasmid-borne ARGs and the bacterial hosts of the corresponding plasmids were determined using the Frucherman-Reingold algorithm as the layout algorithm. A linear regression analysis was performed to assess the correlation between the ARG abundance in plasmids and the corresponding antibiotic consumption. Spearman’s correlation was calculated to evaluate the correlation between the abundance of plasmids harboring ARGs and antibiotic consumption. The Mann-Kendall statistical test was applied to assess the significance of the monotonic increasing trend in ARG abundance.
