# Supplementary material for: Global Increase of Antibiotic Resistance Genes in Conjugative Plasmids
Source: Microbiol Spectr. 2023 Mar 22;11(2):e04478-22. doi: 10.1128/spectrum.04478-22 (PMC10100709; doi:10.1128/spectrum.04478-22)
Supplement: Supplemental file 1 — Supplemental material. Download spectrum.04478-22-s0001.pdf, PDF file, 0.1 MB [file spectrum.04478-22-s0001.pdf]

Supplementary Information for

Global Increase of Antibiotic Resistance Genes in Conjugative Plasmids

Xiaolong Wang,[a] Hanhui Zhang[b], Ximing Xu[c], Hongqiang Ren[b], Daqing Mao[d,*], Pedro J.J. Alvarez[e,*], and Yi Luo,[a,b,*]

[a] College of Environmental Science and Engineering, Ministry of Education Key Laboratory of Pollution Processes and Environmental Criteria, Nankai University, Tianjin 300071, China

[b] State Key Laboratory of Pollution Control and Resource Reuse, School of the Environment, Nanjing University, Nanjing, 210093, China

[c] School of Statistics and Data Science, Nankai University, Tianjin 300071, China

[d] School of Medicine, Nankai University, Tianjin 300071, China

[e] Dept of Civil and Environmental Engineering, Rice University, Houston, Texas 77005, USA

*Corresponding Authors:** Phone: +86 (22) 85358553, E-mail: maodq@nankai.edu.cn, orcid.org/0000-0003-0313-0129

*Corresponding Authors:** Phone: (713) 348-5903, E-mail: alvarez@rice.edu, orcid.org/0000-0002-6725-7199

*Corresponding Authors:** Phone: +86 (22) 85358553, E-mail: luoy@nankai.edu.cn, orcid.org/0000-0001-7707-708X

Table S1 Raw data of plasmid information.

Table S1 is a separated excel file.

Table S2 Co-shared pathogen host (species level) between clinic and natural environment, and the number of connected ARG subtypes.

Genus	Species	Number of connected ARG subtypes
Enterobacter	*Enterobacter cloacae*	21
	Enterobacter hormaechei	5
Shigella	*Shigella sonnei*	6
Escherichia	*Escherichia coli*	59
Klebsiella	*Klebsiella variicola*	17
	Klebsiella oxytoca	13
	Klebsiella pneumoniae	36
Pseudomonas	*Pseudomonas putida*	8
	Pseudomonas aeruginosa	5
Legionella	*Legionella pneumophila*	2
Vibrio	*Vibrio cholerae*	8

Table S3 Raw data for Figure 7 (co-occurrence of plasmid-borne ARGs and bacterial host of the corresponding plasmid, isolated from a clinic or the environment).

Table S3 is a separated excel file.
